# Supplementary material for: Chemical control of the viscoelastic properties of vinylogous urethane vitrimers
Source: Nat Commun. 2017 Mar 20;8:14857. doi: 10.1038/ncomms14857 (PMC5364391; doi:10.1038/ncomms14857)
Supplement: Supplementary Information — Supplementary Figures, Supplementary Tables, Supplementary Note and Supplementary Reference [file ncomms14857-s1.pdf]

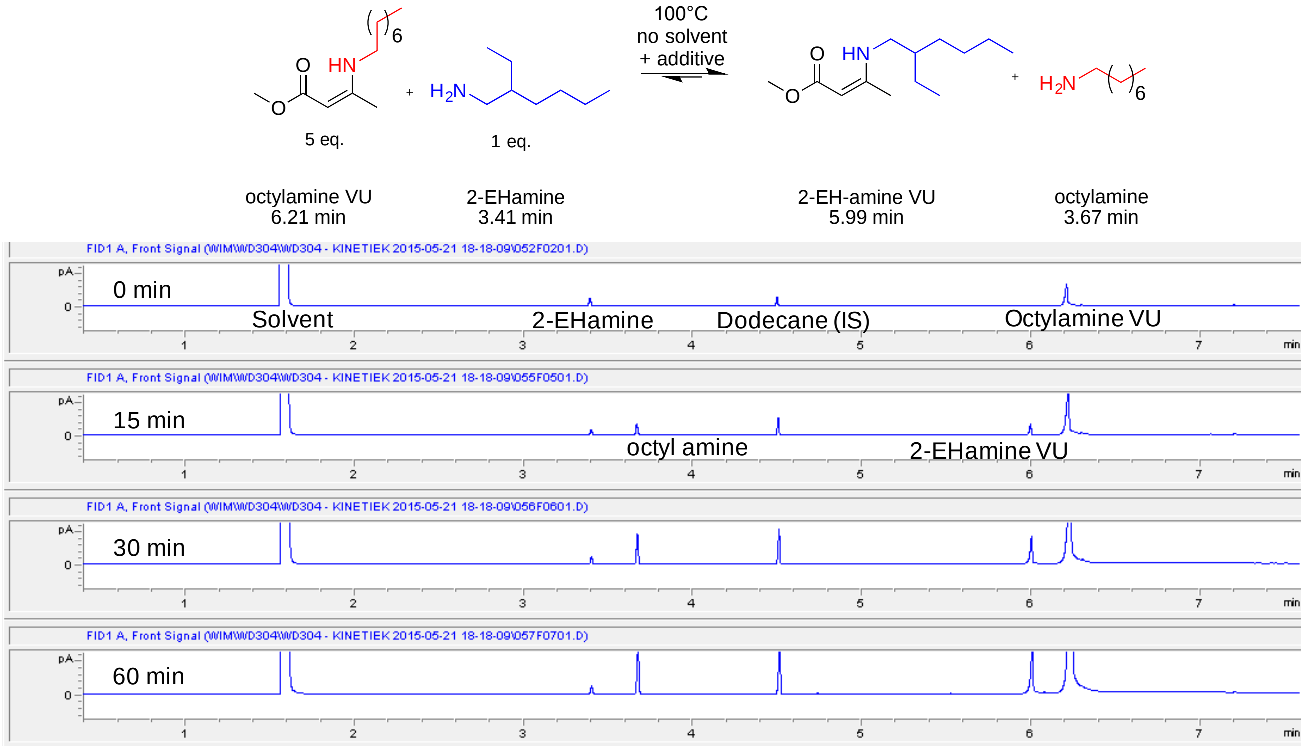

Supplementary Figure 1: GC-traces of the uncatalysed exchange reaction at 100°C, showing the appearance of octylamine (3.56 min) and 2-EHamine VU (5.99 min).

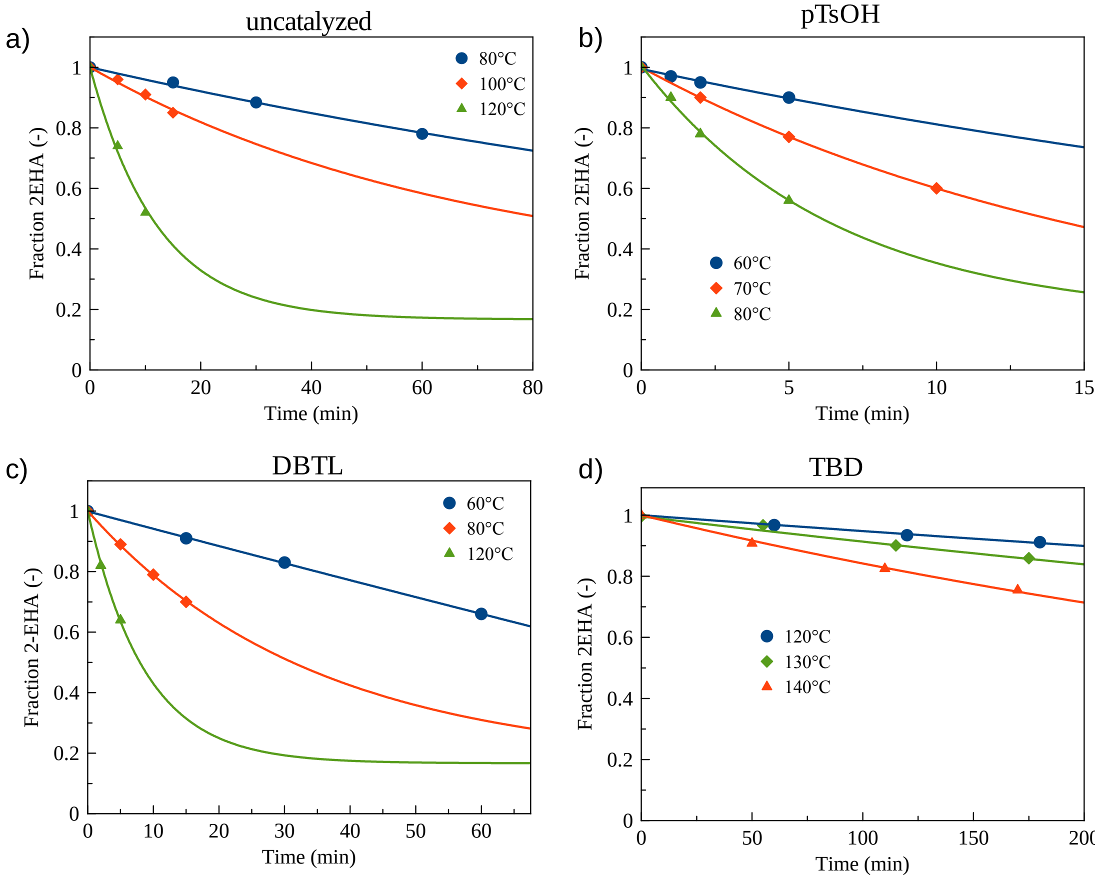

Supplementary Figure 2: Decrease of the fraction of 2-EHA as a function of the time at different temperatures a) without catalyst or in presence of b) *p*TsOH, c) DBTL or d) TBD.

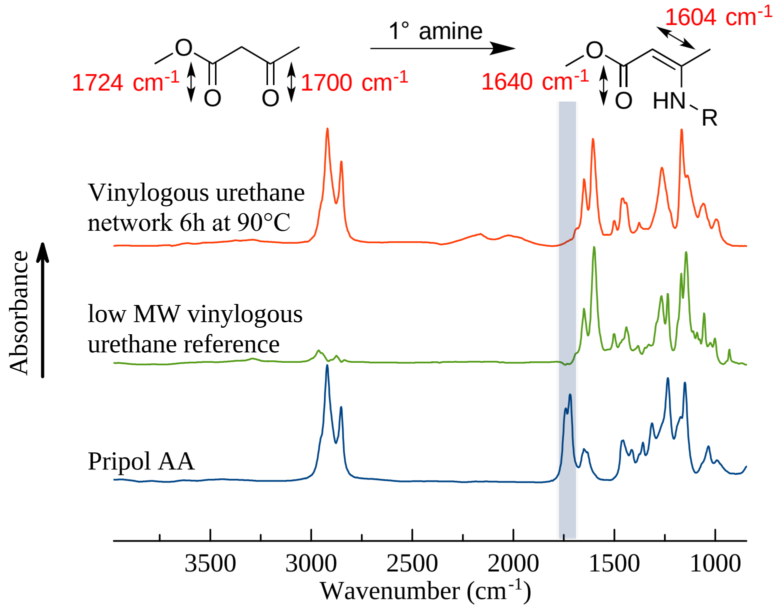

Supplementary Figure 3: FT-IR spectra showing the full conversion of the starting compound (acetoacetylated pripol 2044) to the corresponding vinylogous urethane.

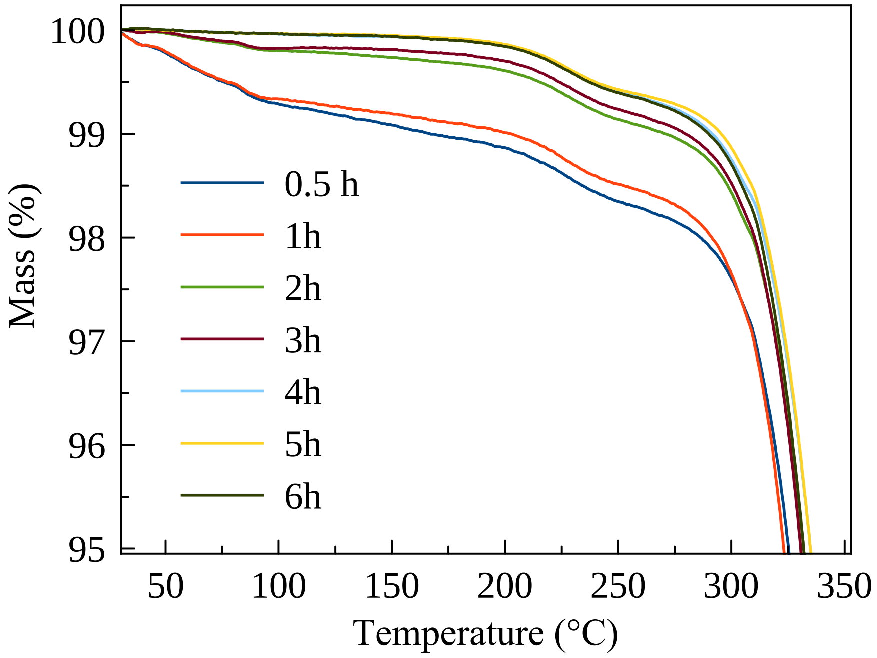

Supplementary Figure 4: TGA measurements of the vinylogous urethane elastomers after different curing times at 90°C. After 4h, no more mass loss is observed before 200°C, indicating the complete evaporation of the water condensate.

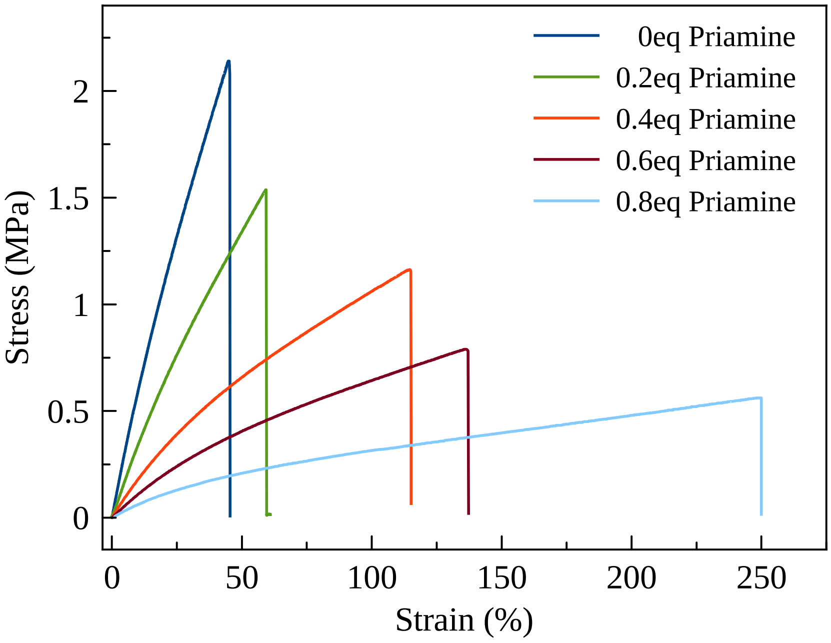

Supplementary Figure 5: Tensile tests of vinylogous urethane vitrimers with different compositions.

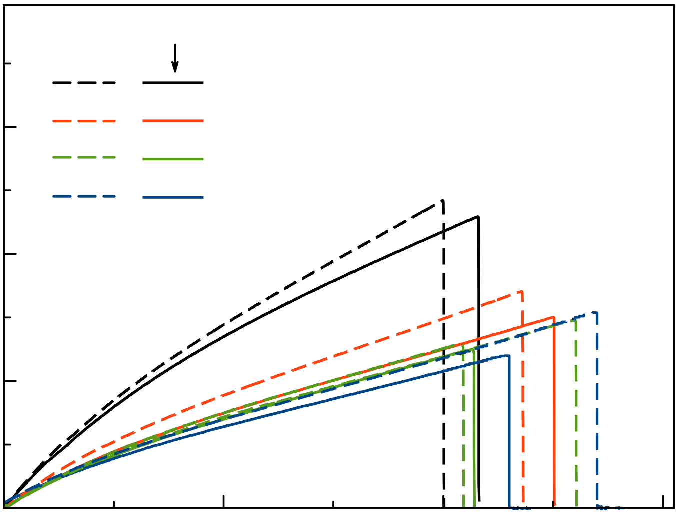

Supplementary Figure 6: Tensile test after recycling of the uncatalyzed (full line) and acid catalyzed (dotted line) samples with a stoichiometric ratio of 0.40 : 0.40 : 0.95. Recycling was performed by first grinding the material followed by compression moulding for 30 minutes at 150°C.

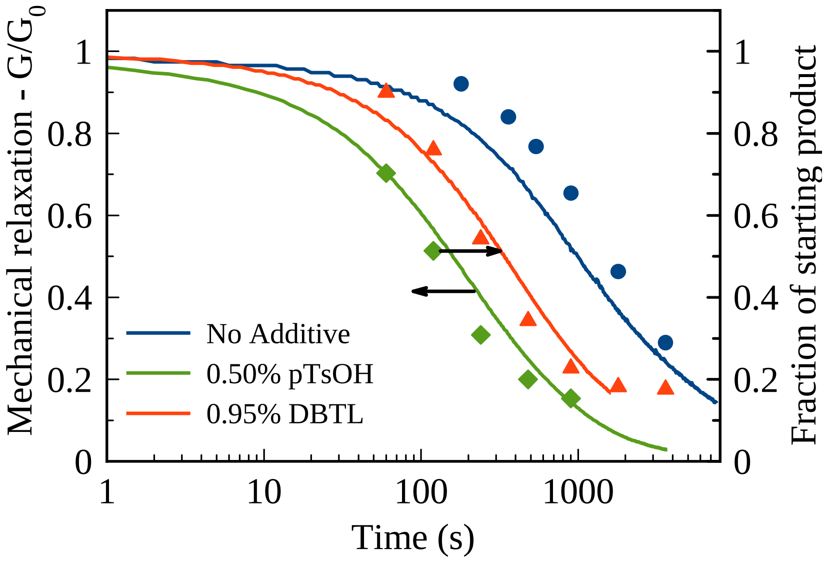

Supplementary Figure 7: Comparison of the conversion of the model compound (dots, right axis) with the mechanical stress-relaxation (full line, left axis) caused by the uncatalyzed, *p*TsOH and DBTL catalyzed exchange reactions, both in the case of model reactions and VU vitrimer samples.

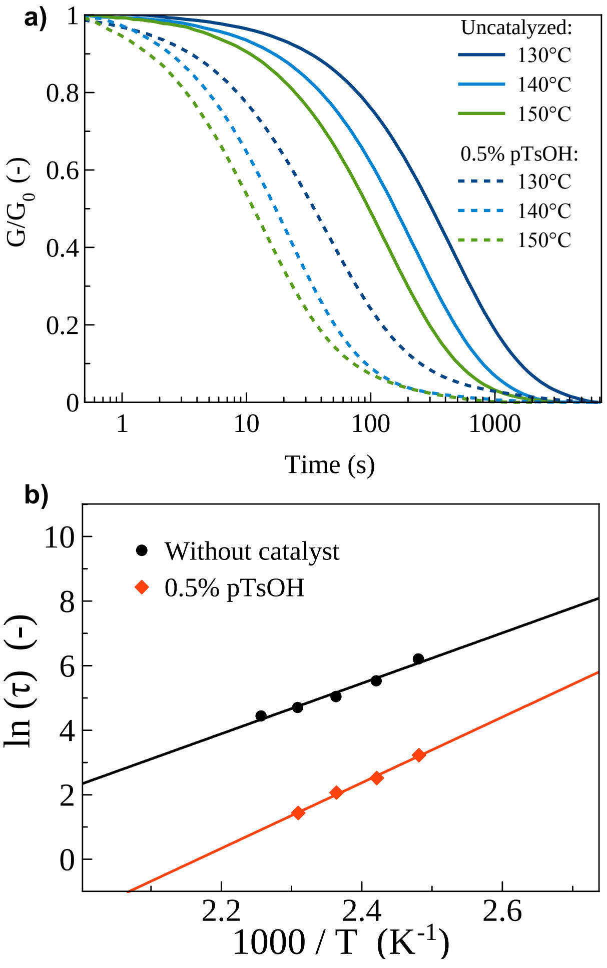

Supplementary Figure 8: Stress-relaxation experiments for rigid VU networks. b) Arrhenius plot of the relaxation times of rigid VU networks.

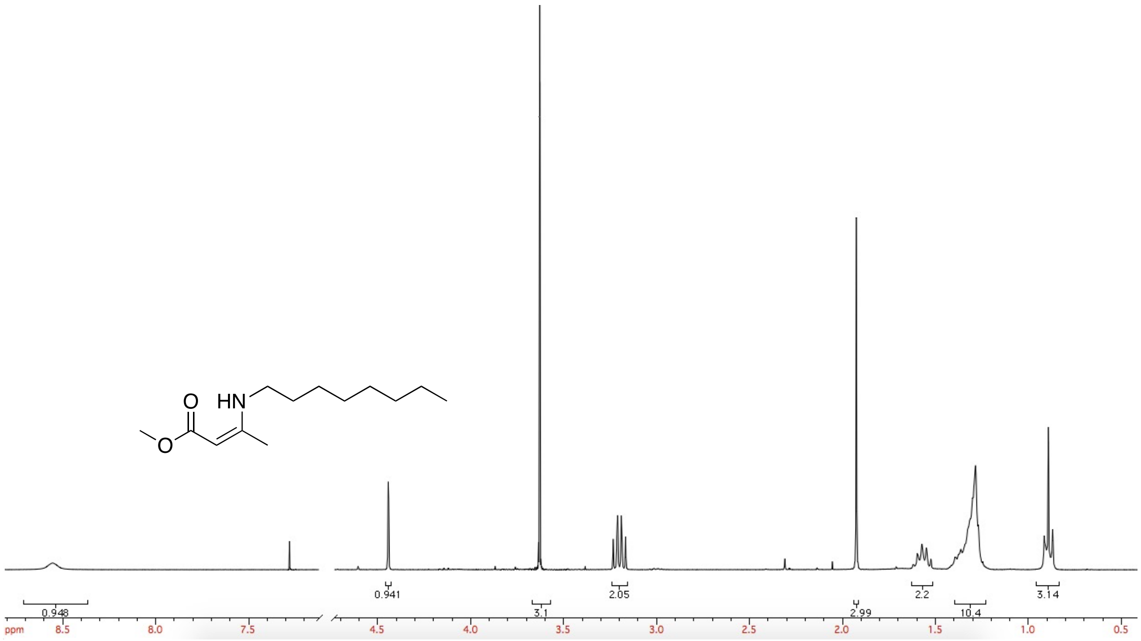

Supplementary Figure 9: ^{1}H-NMR of Methyl-3-(octylamino)but-2-enoate *(i.e. N*-octyl vinylogous urethane).

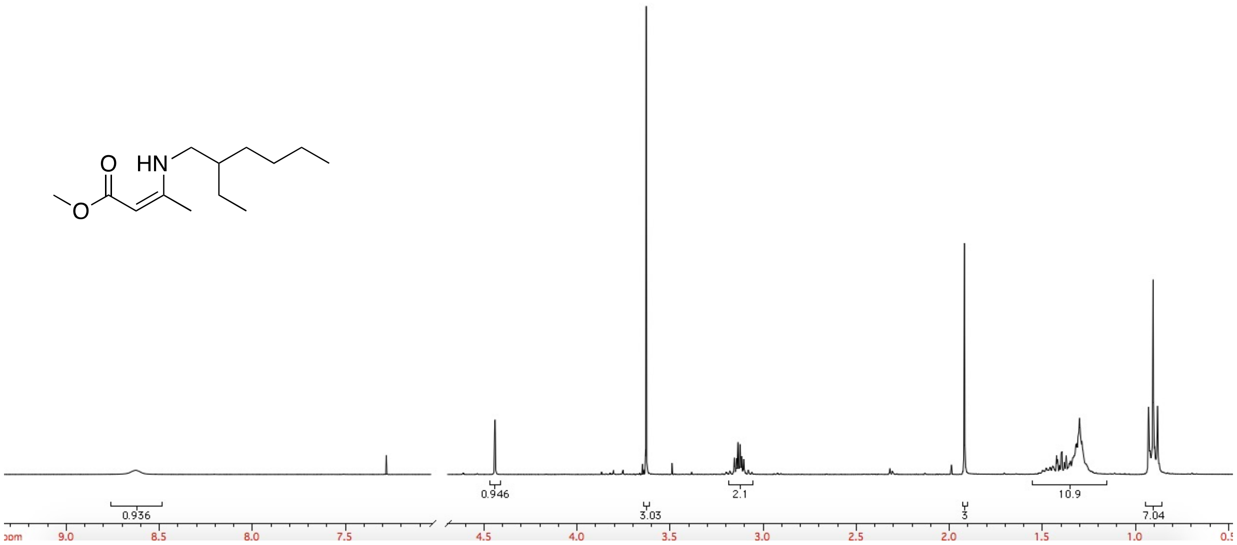

Supplementary Figure 10: ^{1}H-NMR of methyl-3-((2-ethylhexyl)amino)but-2-enoate (N-2EH vinylogous urethane).

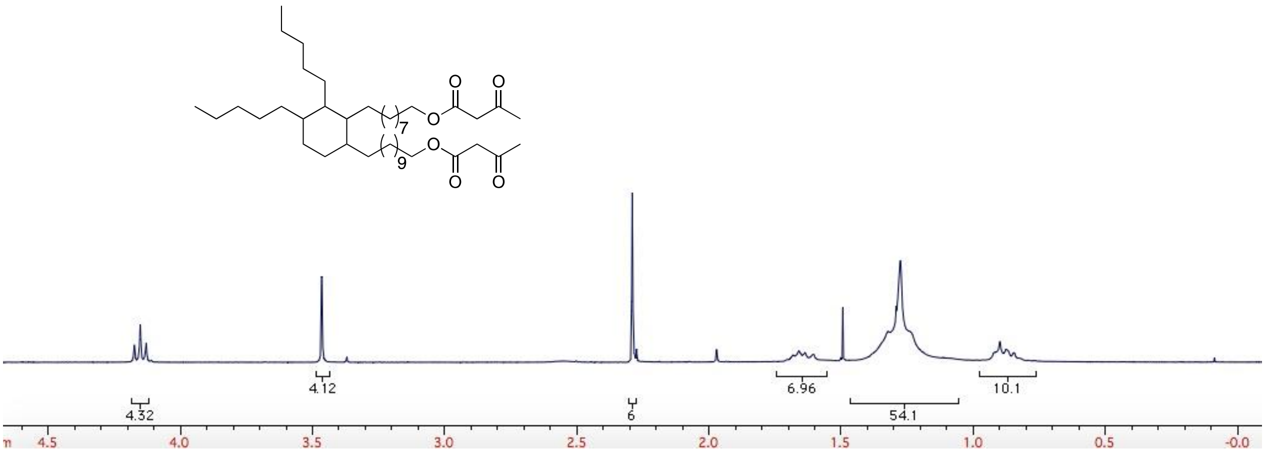

Supplementary Figure 11: ^{1}H-NMR of acetoacetylated pripol 2044.

Supplementary Table 1: k-values obtained from the fitting of the curves of Supplementary Figure 2 and used for the Arrhenius plots.

	Temperature (°C)	k (s^{-1})
Uncatalyzed	80	3,71E-03
	100	9,57E-03
	120	4,80E-02
pTsOH	60	1,93E-02
	70	3,96E-02
	80	8,71E-02
DBTL	60	5,60E-03
	80	2,45E-02
	120	7,11E-02
TBD	120	3,88E-04
	130	8,68E-04
	140	1,75E-03

Supplementary Table 2: Properties of soft VU networks with different compositions.

**Equivalents of 5 : 6 : 7**	T_g **(°C)**	**E' (MPa)[a]**	**Young modulus E (MPa)[b]**	**Elongation at failure (%)[b]**	**Stress at failure (MPa)[b]**
0.00 : 0.67 : 0.95	- 9	3.9	3.35 ± 0.01	46 ± 5	2.22 ± 0.25
0.20 : 0.53 : 0.95	- 20	3.3	2.34 ± 0.02	65 ± 7	1.74 ± 0.27
0.40 : 0.40 : 0.95	- 25	2.2	1.99 ± 0.05	119 ± 9	1.20 ± 0.06
0.60 : 0.27 : 0.95	- 30	1.5	1.13 ± 0.02	140 ± 6	0.81 ± 0.05
0.80 : 0.13 : 0.95	- 33	1.1	0.64 ± 0.05	255 ± 35	0.55 ± 0.03

a) Measured *via* DMTA at 50°C. b) Measured *via* tensile testing, average of 4 measurements.

Supplementary Table 3: Calculated activation energies from the stress-relaxation experiments and calculated freezing transition temperature.

**Catalyst**	**E_a (Kj mol^{-1})**	**T_v (°C)**
Uncatalyzed	81 ± 3	27
0.50% pTsOH	70 ± 4	-1
0.25% H_2SO_4	70 ± 5	10
0.95% DBTL	30 ± 4	-63
1.90% DBTL	31 ± 10	-70
0.50% TBD	122 ± 19	87

Supplementary Note 1

k-values were obtained using[1]:

$$[Reactant] = 1 - (x_{\infty} - \exp\left(\frac{-kt}{x_{\infty}}\right)) \qquad \text{Supplementary Equation 1}$$

With x_{∞} = equilibrium concentration of the products $= \frac{5}{6}$

$Fitted\ for\ k = initial\ rate$

The activation energy was calculated by plotting ln k versus 1000/T.

$$\ln k = -\frac{E_a}{RT} + cte \qquad \text{Supplementary Equation 2}$$

1 Supplementary Reference

1) Logan, S. R. The kinetics of isotopic exchange reactions. *J. Chem. Educ.* **67,** 371 (1990).
